# Effects and Mechanisms of Complementary and Alternative Medicine during the Reproductive Process

**DOI:** 10.1155/2014/698921

**Published:** 2014-04-15

**Authors:** Xiaoke Wu, Ernest Hung Yu Ng, Elisabet Stener-Victorin, Richard S. Legro

**Affiliations:** ^1^Department of Obstetrics and Gynecology National Key Discipline, Specialty and Clinical Trial Base, First Affiliated Hospital, Heilongjiang University of Chinese Medicine, Harbin 150040, China; ^2^Department of Obstetrics and Gynecology, University of Hong Kong, Hong Kong Special Administrative Region, China; ^3^Department of Physiology and Endocrinology, Institute of Neuroscience and Physiology, University of Gothenburg, 40530 Gothenburg, Sweden; ^4^Department of Obstetrics and Gynecology and Department of Public Health Sciences, Penn State University College of Medicine, Hershey, PA 17033, USA

Complementary and alternative medicine (CAM)—which is typically described as being outside of orthodox medicine—consists of a broad spectrum of interventions that aim to promote health and wellbeing and to treat illness. More than 70% of the world's populations rely on some form of CAM for health care, mainly as a compliment to standard care or as a second-line treatment after trying first-line alternatives. The use of CAM is especially common among women of reproductive age, and many of these patients seek out nonmedical treatments and interventions. There have been some benefits reported for women's use of CAM for reproductive health, and recent studies have shown that several different CAM strategies could be beneficial as adjuncts to the conventional medical management of reproductive disorders in women.

Although CAM is widely used among women, strong evidence for its effectiveness is still lacking and the underlying mechanisms behind its effects remain unsolved. The limitations of CAM studies include small sample sizes, nonrandomized or uncontrolled samples, and the self-selected nature of the participants. Thus there is only a low level of support for evidence-based applications of CAM in women with reproductive conditions, and more studies investigating the efficacy, effectiveness, and mechanisms of action of different forms of CAM are needed. We put out a call for papers, and this resulted in the six review articles included in this special issue that cover current CAM therapies for infertility, endometriosis, miscarriage, genital inflammation, and issues associated with delivery.

In this special issue, we have published two reviews describing the evidence that CAM can improve pregnancy rates in subfertile women undergoing* in vitro* fertilization (IVF) and can be beneficial for inducing ovulation in patients with polycystic ovary syndrome (PCOS).* “The effect of complementary and alternative medicine on subfertile women with in vitro fertilization”*, by Y. Zhang et al., describes the current evidence for the effects and mechanisms of Chinese herbal medicine, acupuncture, psychological therapies, temperature therapies, and other CAM therapies on improving the success rate of IVF. PCOS is the most common ovulatory disorder in reproductive-age women.* “Polycystic ovary syndrome: effect and mechanisms of acupuncture for ovulation induction”*, by J. Johansson et al., addresses acupuncture as a potential treatment option for reproductive and endocrine disturbances in women with PCOS. Several studies described in this review indicate that acupuncture is beneficial in treating the ovulatory dysfunction associated with PCOS.

CAM has long been regarded as an effective and safe way to decrease the risk of miscarriage and to maintain a successful pregnancy, and Chinese herbal medicine has become a popular and common form of CAM for the prevention of miscarriages. In* “Systematic review of Chinese medicine for miscarriage during early pregnancy”*, L. Li et al. have identified 39,792 relevant pieces of literature for review and they summarize the clinical applications of Chinese herbal medicine during pregnancy. Based on this evidence, they suggest that Chinese herbal medicine combined with Western medicine can be effective in preventing miscarriage and relieving its clinical symptoms, but Chinese herbal medicine alone might not be effective. Rigorous scientific and clinical studies are necessary, therefore, to confirm the effectiveness of Chinese herbal medicine.

In S. Kong et al.'s review* “The complementary and alternative medicine for endometriosis: a review of utilization and mechanism”*, they assess the role of CAM on endometriosis and provide evidence from a number of clinical and experimental studies for both the therapeutic efficacy of CAM and possible mechanisms for these effects. The CAM therapy for endometriosis includes herbs, acupuncture, microwave-physiotherapy, Chinese herbal medicine enema, and psychological interventions. These CAM therapies are effective at relieving dysmenorrhea, shrinking adnexal masses, and improving pregnancy rates with fewer unpleasant side effects compared to standard hormonal and surgical treatments.

Genital infection is a very common gynecological condition. In* “Applications and therapeutic actions of complementary and alternative medicine for women with genital infection”*, C. Liu et al. review the current progress of using CAM therapies to treat genital infections such as vulvitis, vaginitis, cervicitis, and pelvic inflammatory disease. They also introduce traditional Chinese medicine, psychological intervention, and physical therapy as treatments for endometriosis.

*“Analyzing the study of using acupuncture in delivery in the past ten years in China”*, by Y. Chen et al., provides a review of 87 articles published from 2002 to 2012. Clinical research indicates that acupuncture relieves labor pain, induces maternal uterine contractions, shortens the birthing process, and treats postpartum complications. Preclinical research has found that acupuncture can modulate certain hormones and improve uterine contractions in late-stage pregnant rats.

Despite the evidence provided in these reviews, large, multicenter, and well-designed randomized controlled trials (RCTs) are needed to evaluate the effectiveness and safety of CAM treatments for human reproductive issues. It is important to have more RCTs that are performed following the standards for reporting interventions in clinical trials and fewer commercially driven RCTs, especially CAM clinical trials. These clinical trials should be reported in accordance to the Consolidated Standards of Reporting Trials (CONSORT) protocol in the various areas of reproduction. This would improve the reporting of RCTs and make it easier to understand a trial's design and conduct and to assess the validity of its results.

